# Universal Behavior in Enantioselective Adsorption of Amino Acids onto Chiral Terbium Phosphate Nanocrystals

**DOI:** 10.3390/molecules31183187

**Published:** 2026-09-10

**Authors:** Abdullah Idrees, Gil Markovich

**Affiliations:** 1 School of Chemistry, The Raymond and Beverly Sackler Faculty of Exact Sciences, Tel Aviv University, Tel Aviv 6997801, Israel; 2 Department of Materials Science and Engineering, The Iby and Aladar Fleischman Faculty of Engineering, Tel Aviv University, Tel Aviv 6997801, Israel

**Keywords:** chiral surfaces, nanocrystals, enantioselective adsorption, amino acids, enantiomeric excess

## Abstract

The homochirality of biomolecules motivates the search for chiral surfaces capable of enantioselective recognition, yet systematic links between amino acid functional-group geometry and adsorption on chiral inorganic nanocrystals (NCs) remain limited. Here we investigate the enantioselective adsorption of asparagine, serine, and histidine onto intrinsically chiral Λ- and Δ-TbPO_4_·H_2_O NCs, which crystallize in a monoclinic (pseudo-hexagonal) structure. Individual-enantiomer adsorption isotherms and enantiomeric excess (*ee*) from racemic mixtures were determined using circular dichroism spectroscopy combined with potentiometric titration. Adsorption of single enantiomers followed the Langmuir model at low coverage, with L-enantiomers preferring Λ-NCs and D-enantiomers preferring Δ-NCs; matched chiral pairs showed roughly twofold higher equilibrium constants than mismatched pairs. At relatively high surface coverage, the adsorption behavior deviated from the Langmuir model and fitted the Frumkin model, which contains lateral attraction between adsorbed molecules. Enantioselectivity was highest at low racemate concentrations and declined with increasing total racemate concentrations, mirroring trends previously found for tartaric acid and aspartic acid on the same NCs. Tb^3+^–Tb^3+^ spacings on the dominant facets closely matched intramolecular functional-group distances in the amino acids, supporting a three-point chiral recognition mechanism.

## 1. Introduction

The homochirality of biomolecules, including amino acids, carbohydrates, proteins, and DNA, is a fundamental characteristic of life on Earth [1,2]. Due to the homochiral nature of biological systems, the two enantiomers of a chiral molecule can exhibit significantly different interactions and physiological activities [3,4,5]. This difference has generated considerable demand for enantiomerically pure compounds, particularly in the pharmaceutical and biochemical industries, where the global market for chiral molecules represents a major economic sector [6]. Consequently, the development of efficient enantioselective approaches for molecular recognition, separation, and asymmetric catalysis has attracted extensive scientific and technological interest.

Although bulk metals commonly possess achiral crystal structures, intrinsically chiral metal surfaces can be generated by exposing low-symmetry, high-Miller-index crystal planes [7,8,9]. These surfaces contain terrace–step–kink arrangements that lack mirror symmetry and exist as two non-superimposable mirror-image structures. Such well-defined surfaces have been extensively used as model systems to study fundamental aspects of chiral recognition, including enantiospecific adsorption, desorption kinetics, molecular orientation, surface reactions, and electrochemical behavior [10,11,12,13,14,15,16,17,18,19,20,21,22,23,24].

Single-crystal metal surfaces have provided valuable understanding of the relationship between atomic surface structure and enantioselective molecular interactions [25,26,27,28]. Studies on naturally chiral high-index copper surfaces demonstrated that the spatial arrangement of surface atoms strongly influences adsorption preference between molecular enantiomers [29,30]. Investigations involving amino acids, such as aspartic acid, revealed that enantioselective adsorption originates from differences in adsorption affinity, molecular organization, and cooperative interactions between adsorbed species [30,31].

Although chiral minerals have been investigated as possible contributors to the origin of biological homochirality, many systems, such as quartz, display relatively weak amino acid enantioselectivity due to limited surface interactions and the presence of multiple exposed crystal facets [32]. Other inorganic materials with chiral terminations of achiral crystals, including calcite, have demonstrated chiral recognition capabilities [33,34,35]. More recently, advanced inorganic systems have shown enhanced enantioselective adsorption behavior [36]. However, systematic studies of amino-acid-dependent enantioselective adsorption on intrinsically chiral inorganic NC surfaces, particularly with respect to the role of functional group geometry and molecular structure, remain limited.

This limitation has stimulated growing interest in intrinsically chiral inorganic materials that offer more robust and stable chiral environments. Chiral surfaces play a crucial role in controlling the interactions between chiral molecules and solid materials, offering promising platforms for enantioselective adsorption, molecular recognition, and heterogeneous catalysis. Traditionally, chiral interfaces have been prepared by modifying achiral inorganic surfaces with enantiomerically pure organic molecules, where adsorbed chiral modifiers induce surface chirality and promote enantiospecific interactions with target molecules [37,38,39,40,41,42,43]. However, the limited chemical and thermal stability of organic modifiers restricts their applicability, particularly under demanding reaction conditions.

Among more robust alternatives, intrinsically chiral NCs are especially attractive because they combine high surface area, structural chirality, and well-defined surface properties. Recently, our group developed enantiopure TbPO_4_·H_2_O NCs, which crystallize in a chiral space group, through tartaric acid-directed growth [44,45,46,47]. The dominance of a single low-Miller-index (100) surface facet type creates a uniform chiral inorganic interface, making these NCs an excellent platform for investigating fundamental mechanisms of enantioselective adsorption. These NCs exhibited relatively high enantioselectivity towards adsorption of tartaric acid and aspartic acid molecules from racemic mixtures, which increased with the lowering of surface coverage by the molecules [48].

**Scheme 1:**
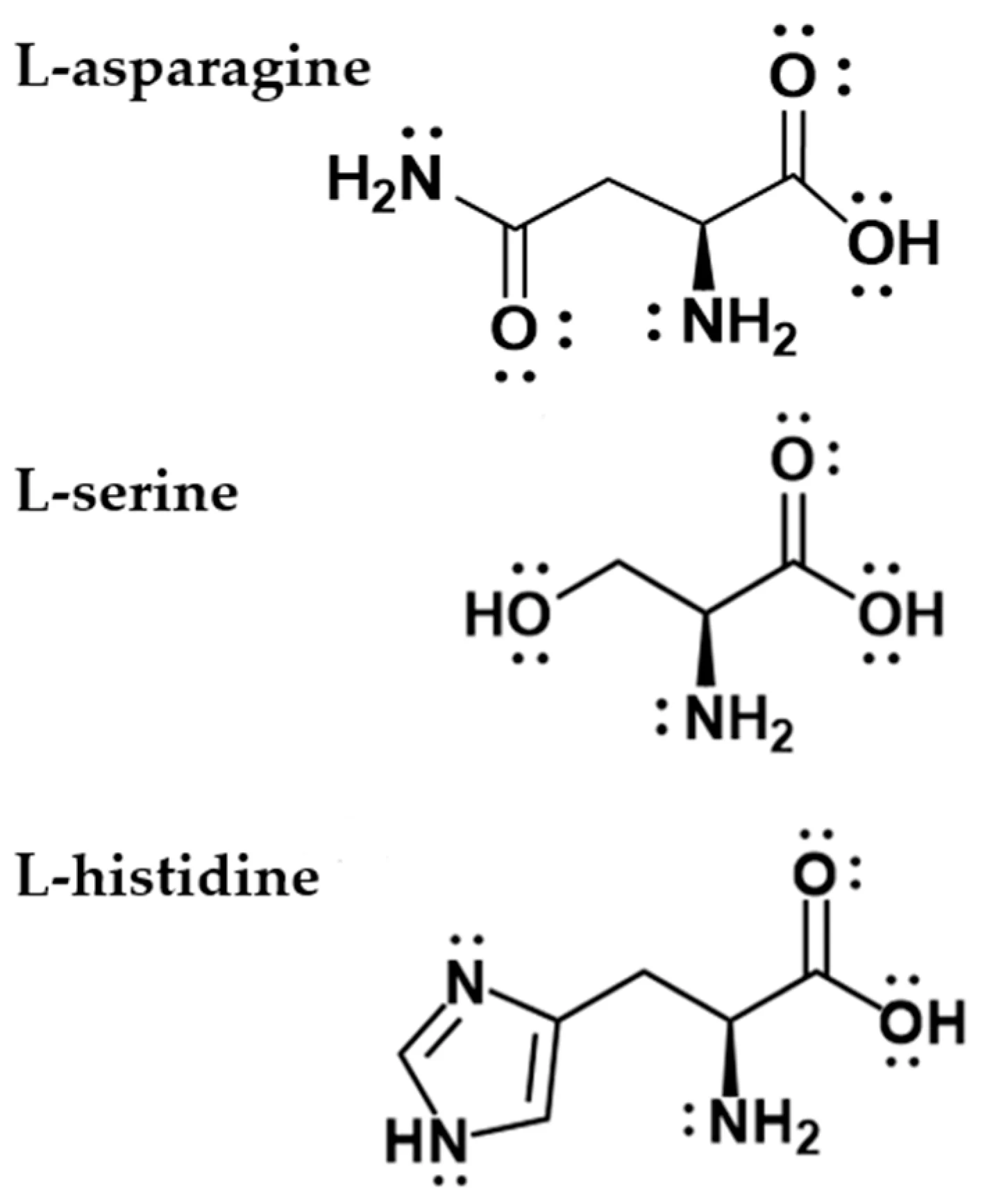
Chemical structures of L-Asn, L-Ser, and L-His amino acids used to investigate enantioselective adsorption on chiral TbPO_4_·H_2_O NC surfaces.

In the present study, we investigate the enantioselective adsorption of the amino acids asparagine (Asn), serine (Ser), and histidine (His) on the intrinsically chiral surfaces of Λ- and Δ-TbPO_4_·H_2_O NCs, extending our previous work [48] to three amino acids not yet examined. These molecules were chosen to probe how side-chain functionality governs chiral recognition: they bear structurally distinct coordinating groups, including carboxylate, amino, amide, hydroxyl, and imidazole, whose spatial arrangement enables multi-point binding with the chirally organized Tb^3+^ surface sites (Scheme 1). CD spectroscopy was used to quantify enantioselective adsorption of individual enantiomers and racemic mixtures, while potentiometric titration independently determined total adsorbed quantities, together providing enantiomeric excess (*ee*) values inaccessible by CD alone.

## 2. Results

### 2.1. Enantioselective Adsorption of Individual Amino Acid Enantiomers

The adsorption behavior of single enantiomers of Asn, Ser, and His on chiral Λ- and Δ-TbPO_4_·H_2_O NC surfaces was systematically investigated using CD spectroscopy. Concentration calibration curves established from CD measurements of enantiopure amino acid solutions in water enabled quantitative determination of each enantiomer concentration in the supernatant following adsorption (Supplementary Information, Figure S1). In each experiment, solutions of either L- or D-Asn, L- or D-Ser, or L- or D-His at selected initial concentrations were incubated with a fixed quantity of Λ- or Δ-TbPO_4_·H_2_O NCs. After equilibration, the NC–adsorbate complexes were isolated by centrifugation, and the CD signal of the remaining supernatant was used to determine the equilibrium free amino acid concentration. The adsorbed equivalent concentration (*C_ads_*) was then obtained from the difference between the initial (*C_initial_*) and equilibrium free molecule (*C_free_*) concentrations, according to Equation (1):

C_*ads*_ = C_*initial*_ − C_*free*_ (1)

### 2.2. Asparagine, Serine, and Histidine: Frumkin vs. Langmuir Adsorption Models

The adsorption isotherms for individual Asn, Ser, and His enantiomers for relatively low surface coverages on Λ- and Δ-TbPO_4_·H_2_O NCs are presented in Figure S2. In all cases, the adsorbed equivalent concentration increased progressively with rising equilibrium free amino acid concentration in solution. A pronounced enantioselective trend was consistently observed: L-Asn, L-Ser, and L-His adsorbed more strongly on Λ-NCs, while D-Asn, D-Ser, and D-His were preferentially adsorbed on Δ-NCs. The opposite chiral combinations of L-enantiomers on Δ-NCs and D-enantiomers on Λ-NCs yielded systematically lower adsorbed concentrations across the entire concentration range studied.

The experimental adsorption isotherms for Asn, Ser, and His were well described by the Langmuir adsorption model at the initial concentration range ≤ 5 mM:

(2) $$C_{ads}=Q\frac{KC_{free}}{1+KC_{free}}$$

where K is the adsorption equilibrium constant and Q is the maximal adsorption capacity in equivalent concentration units. The Langmuir model fits are shown as solid lines in Figure S2 and demonstrate good agreement with the experimental data for single-enantiomer systems.

This mirror-image adsorption selectivity directly reflects the chiral symmetry of the NC surfaces and confirms that stereospecific interactions between the amino acid functional groups and the chirally organized Tb^3+^ binding sites govern the adsorption preference.

**Figure 1:**
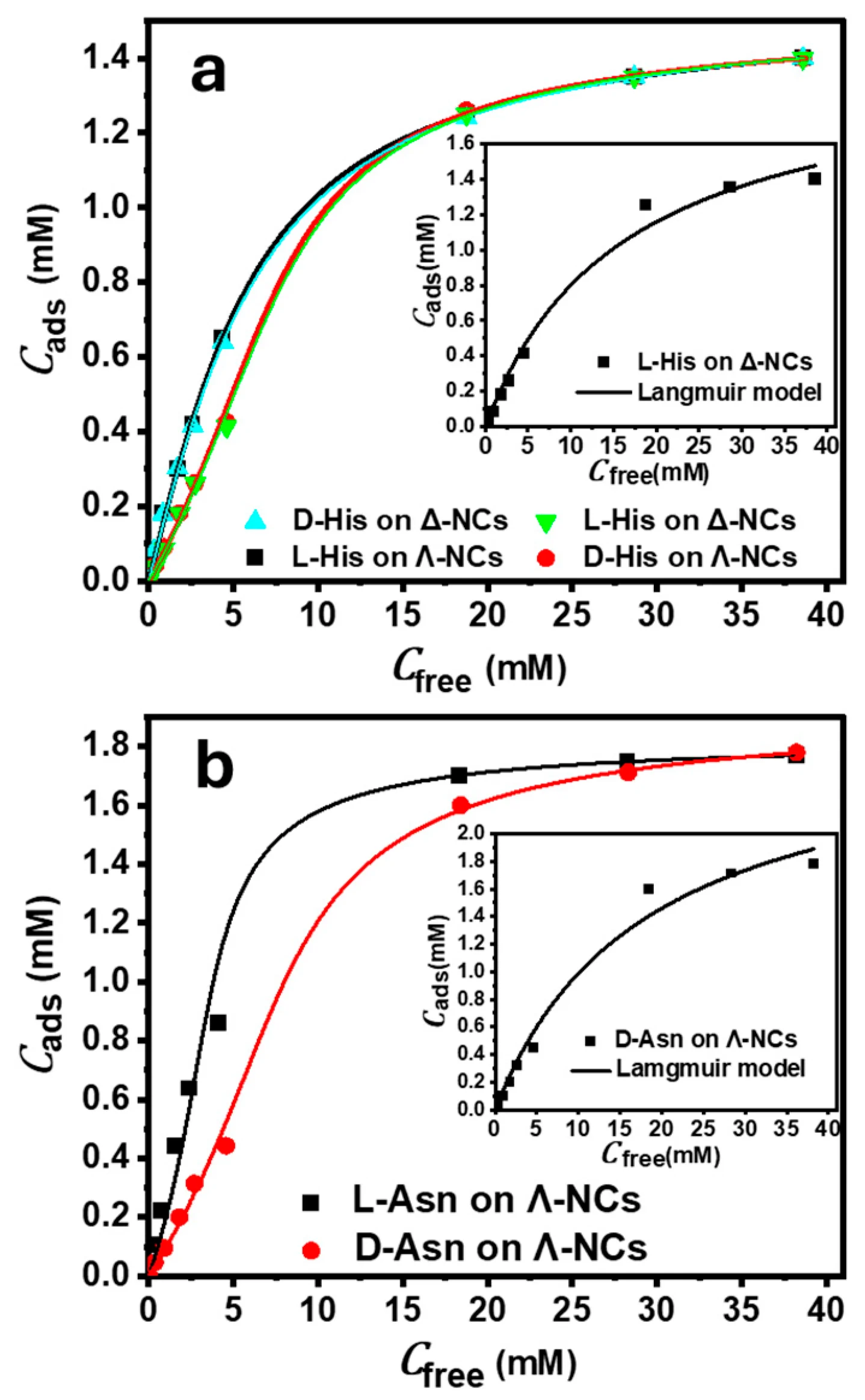
(**a**) The adsorption of single His enantiomers (in terms of equivalent adsorbed concentration) to Λ- and Δ-NCs as a function of the free His enantiomer concentration. The symbols show experimental data, whereas the lines represent the fitted Frumkin model curves. (**b**) The same for Asn. The insets show attempted fit to a Langmuir model for one representative combination. Similar mismatch to the Langmuir model was observed for all 4 combinations.

We have extended the measured adsorbed concentration range to higher surface coverage values for the His and Asn systems, as shown in Figure 1. As seen in the two graph insets, the Langmuir model significantly fails at higher concentrations, as the adsorbed molecules approach monolayer saturation. We have found that the Frumkin adsorption model fits best the experimental points:

(3) KCfree=CadsQ−Cadse−UCadsQ

where the parameter U describes the lateral attraction between the adjacent adsorbed molecules (in units of $k_{B}T$). At low coverage (small $C_{ads}$), the expression converges to the Langmuir model (as seen in Figure S2 and insets in Figure 1).

The adsorption equilibrium constants K and maximum adsorption capacities Q extracted from the low-concentration (≤5 mM) Langmuir model fits are summarized in Table 1. For all three amino acids, the equilibrium constants corresponding to the preferred chiral pairings (L-enantiomers on Λ-NCs and D-enantiomers on Δ-NCs) were approximately two-fold greater than those of the non-preferred configurations (L-enantiomers on Δ-NCs and D-enantiomers on Λ-NCs), providing quantitative evidence for pronounced enantioselective adsorption on the chiral NC surfaces. The maximum adsorption capacity Q, however, followed a different trend depending on the amino acid. Comparable Q values were obtained Asn, aspartic acid (Asp), and tartaric acid (TA) [48], while relatively high Q was obtained for Ser adsorption and low for His, which makes sense due to the relatively small size of the former and larger size of the latter.

The value of the adsorption equilibrium constant does not vary systematically among Ser, Asn, and His. Ser, bearing a hydroxyl group, exhibited the weakest adsorption affinity among the three amino acids. This behavior closely parallels that previously observed for TA, where hydroxyl and carboxyl groups are the sole coordinating functionalities, yielding comparably low K values relative to Asp (see Table 1). This analogy consistently points to the conclusion that hydroxyl coordination to Tb^3+^ sites is inherently weaker than that of carboxyl, amine or amide groups.

Similar conclusions can be drawn from the fits to the Frumkin isotherm for His and Asn for the larger concentration range, as presented in Table 2. While the fitted adsorption constants are 20–30% smaller than those obtained from the Langmuir model at the low concentrations, the same trends are preserved, and there is still a factor ~2 difference in adsorption constant between the matching pairs of Λ-L/Δ-D and non-matching ones, Λ-D and Δ-L. Regarding the inter-molecular interaction energy, U, we note that for His, the non-matching pairs have an interaction energy ~×2.5 larger than the matching ones, which may indicate different adsorption configurations in these two cases, leading to significantly different hydrogen bonding states in the two configurations. However, for Asn, those energies are about the same, perhaps showing that this molecule does not significantly change adsorption configuration in the two adsorption states.

Asn, with its amide-containing side chain (–CH_2_CONH_2_) in addition to the amino and carboxyl groups [49,50], provides multiple hydrogen bond donors and acceptors as well as additional coordination sites for interaction with the chirally organized Tb^3+^ ions at the NC surface, resulting in a stronger adsorption relative to Ser. His exhibited the strongest adsorption affinity of the series, attributed to the imidazole ring, which offers a nitrogen-rich coordination environment that interacts more effectively with surface Tb^3+^ sites.

The exact nature of the coordination of the various functional groups to the Tb^3+^ sites is not completely clear. In all cases, pH ≥ 5.5 dictates that the carboxylate groups are deprotonated, and thus the negatively charged oxygen atoms would probably both be attracted to the Tb^3+^, with the exact configuration to be determined by quantum chemical calculations (notoriously difficult for lanthanides). Carboxylate–lanthanide ion interaction is considered the strongest coordination interaction of lanthanides [51], as their interactions with ligands are primarily electrostatic in nature.

The amine groups, while protonated when free in solution at the pH values of the adsorption experiments (5.5–6.7), must lose the proton upon coordination with the Tb^3+^. We expect this to be the case in all three amino acids studied here. Then, there is the state of coordination of the different side groups. The relatively weaker interaction of Ser’s hydroxyl with the surface indicates that it does not lose the proton and the oxygen’s lone pairs (weakly) interact with the cation. In the case of Asn, it is hard to tell how the amide group interacts with the Tb^3+^ sites and again, quantum chemical calculations would be required to better understand this coordination. Last, the imidazole ring of His at the pH of the adsorption experiment (6.7) is expected to be neutral and can thus interact with the Tb^3+^ sites [52]. His has the highest adsorption constant of the three amino acids discussed in this work, and slightly lower than that of aspartic acid [48]. This probably indicates that the neutral primary amine groups have a stronger coordination strength to the surface Tb^3+^, followed by the imidazole nitrogen(s) and by an amide group. In contrast to the very strong interaction of imidazole with transition metals, they exhibit more modest interaction with lanthanide ions [53].

### 2.3. Enantioselective Adsorption of Racemic Amino Acid Mixtures on the Chiral Surface of TbPO_4_·H_2_O NCs

**Figure 2:**
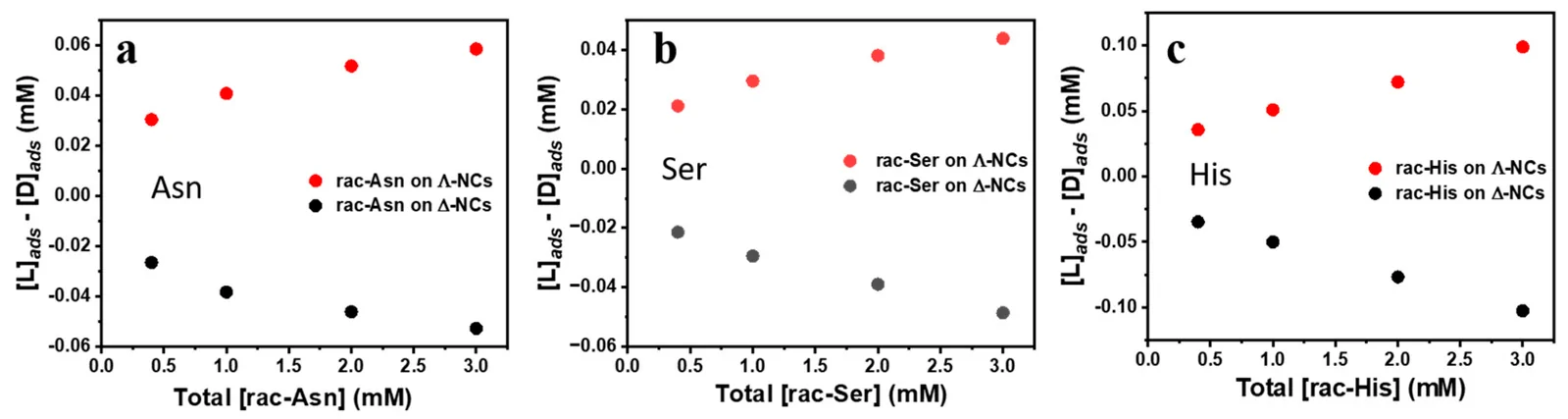
Enantioselective adsorption of racemic amino acid mixtures on chiral Λ- and Δ-TbPO_4_·H_2_O NCs. The difference between the adsorbed equivalent concentrations of the two enantiomers ([L]*_ads_* − [D]*_ads_*) is displayed as a function of the total initial concentration of the racemic amino acid in solution. (**a**) Asn; (**b**) Ser; and (**c**) His.

The enantioselective adsorption from racemic mixtures of asparagine (rac-Asn), serine (rac-Ser), and histidine (rac-His) on the chiral surfaces of Λ- and Δ-TbPO_4_·H_2_O NCs was investigated by CD spectroscopy and potentiometric titration. Racemic amino acid mixtures were added to suspensions containing either Λ-NCs or Δ-NCs, and after equilibration, the supernatant containing the free non-adsorbed molecules was isolated by centrifugation prior to CD measurement. CD spectroscopy cannot directly quantify the amount of each adsorbed enantiomer; instead, the observed CD signal in the supernatant reflects the difference between the concentrations of the two enantiomers in solution, which carries the opposite sign to the equivalent concentration difference of the molecules adsorbed on the NC surface. As shown in Figure 2, the enantiomers’ equivalent concentration difference at the NC surface increased with increasing total racemate concentration for both Λ- and Δ-TbPO_4_·H_2_O NCs.

For rac-Asn (Figure 2a), the adsorption differential [L-Asn] − [D-Asn] increased progressively with total racemate concentration on Λ-NCs, while the opposite trend was observed on Δ-NCs, demonstrating the preferential adsorption of opposing enantiomers on each chiral surface. A qualitatively similar but quantitatively reduced enantioselective response was observed for rac-Ser (Figure 2b), with Λ- and Δ-TbPO_4_·H_2_O NCs again displaying opposing adsorption preferences across the concentration range examined. The absolute magnitude of the adsorption differential [L-Ser] − [D-Ser] was consistently smaller than that recorded for Asn at similar concentrations, in agreement with the lower Langmuir adsorption equilibrium constants determined for Ser. Adsorption from rac-His (Figure 2c) followed the same opposing-preference pattern on Λ- and Δ-NCs, but with the largest adsorption differential [L-His] − [D-His] among the three amino acids, reaching approximately +0.10 mM on Λ-NCs and −0.11 mM on Δ-NCs at 3 mM total racemate concentration.

### 2.4. Enantioselective Adsorption and Enantiomeric Excess

To quantify the *ee* arising from racemate adsorption, the total adsorbed equivalent concentrations of His and Asn were measured in each experiment with varying amino acid racemate concentration (Ser was ignored due to weaker enantioselective adsorption):

(4) $$ee\left(\%\right)=100\times \left|\frac{{\left[L\right]}_{ads}-{\left[D\right]}_{ads}}{{\left[L\right]}_{ads}+{\left[D\right]}_{ads}}\right|$$

This concentration was determined by NaOH titration of the free amino acids remaining in solution after separating out the NCs carrying the adsorbed molecules by centrifugation (see Experimental Methods and Supplementary Information Figure S3 and Table S1). The difference between the adsorbed equivalent concentrations of the two enantiomers shown in Figure 2a,c was then divided by the estimated total adsorbed equivalent concentration, calculated by subtracting the free total amino acid concentration determined by titration from the initial total amino acid concentration.

**Figure 3:**
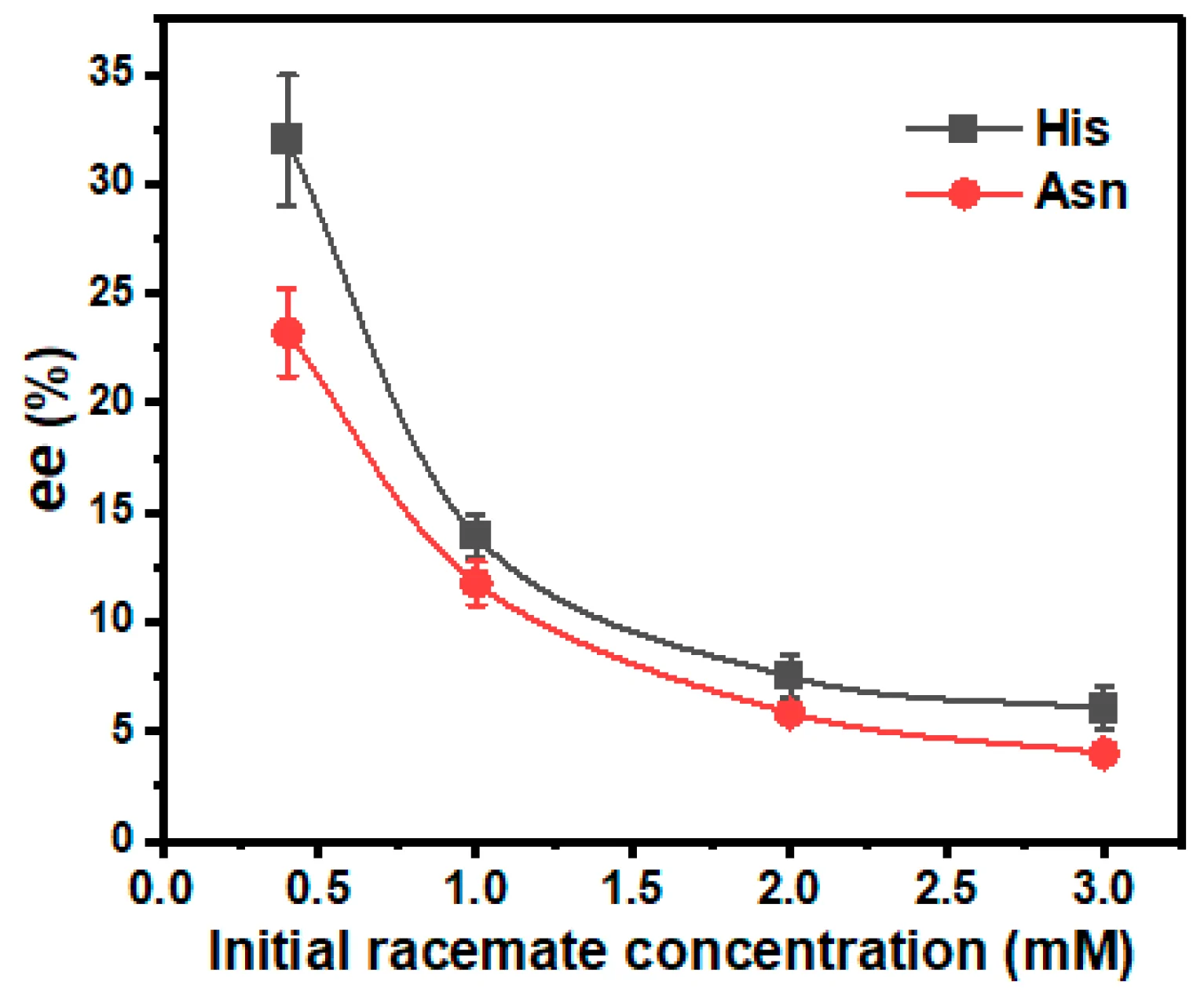
*ee* of surface coverage resulting from rac-His and rac-Asn adsorption on enantiopure TbPO_4_·H_2_O NCs as a function of initial racemate concentration. Error bars represent the uncertainty associated with the determination of adsorbed quantities from the titration analysis. Solid lines are drawn as guides to the eye.

Figure 3 presents the adsorbed *ee* values obtained from rac-His and rac-Asn adsorption experiments, which can be directly compared to those previously reported for rac-ASP and rac-TA [48]. For rac-His, the *ee* reached approximately 32% at 0.4 mM before declining rapidly to approximately 6% at 3 mM, while rac-Asn exhibited a lower initial *ee* of approximately 22.5% at 0.4 mM, similarly decreasing to approximately 4% at 3 mM. This concentration-dependent behavior is consistent with that previously observed for rac-ASP and rac-TA, where the *ee* climbed to approximately 42% and 14% at 0.4 mM, respectively, before declining sharply to approximately 8% and 1% at 3 mM. Across all four molecules, a universal trend is evident: *ee* discrimination is most pronounced at low racemate concentrations and diminishes progressively with increasing concentration, reflecting the gradual saturation of enantioselective binding sites on the NC surface. The overall ranking of *ee* follows the order TA < Asn < His < ASP, apparently consistent with the hierarchy of the side chain’s functional group coordination capacity (hydroxyl < amide < imidazole < carboxyl) toward the chirally organized Tb^3+^ ion sites.

It should be noted that this trend of increased *ee* with lowering surface coverage violates the Langmuir model extended for two competing molecular species. In that model, the adsorbed enantiomer ratio should be proportional to the free solution enantiomer ratio at all concentrations:

(5) $$\frac{C_{ads}^{L}}{C_{ads}^{D}}=\frac{K_{L}}{K_{D}}\frac{C_{free}^{L}}{C_{free}^{D}}$$

where $C_{ads}^{L},\;\;C_{ads}^{D}$ are the adsorbed equivalent concentrations of the two enantiomers of a particular chiral molecule, $C_{free}^{L},\;{C\;}_{free}^{D}$ are the corresponding free molecule concentrations in solution and $K_{L},\;K_{D}$ are the corresponding adsorption equilibrium constants. Such an expression should not exhibit a strong total concentration dependence, unlike the results in Figure 3. Hence, while single enantiomer adsorption seems to fit the Langmuir model at low concentrations, it appears that in the case of adsorption from a racemic mixture, it does not follow the model, probably due to the existence of lateral intermolecular (inter-enantiomer) interactions.

This phenomenon can be rationalized by considering the availability of adsorption sites per molecule at low concentrations [48]. Under dilute conditions, each molecule can maximize multidentate interactions of its functional groups—carboxyl, amino, and amide/imidazole side chain—with the accessible surface sites (primarily Tb^3+^) [48,54]. At higher concentrations, surrounding adsorption sites become increasingly occupied by neighboring molecules where cooperative interactions between same or opposite enantiomers adsorbed at adjacent sites become progressively more significant, thereby diminishing the net enantioselectivity [48].

### 2.5. Geometric Matching Between Amino Acid Functional Groups and Surface Tb^3+^ Sites

**Figure 4:**
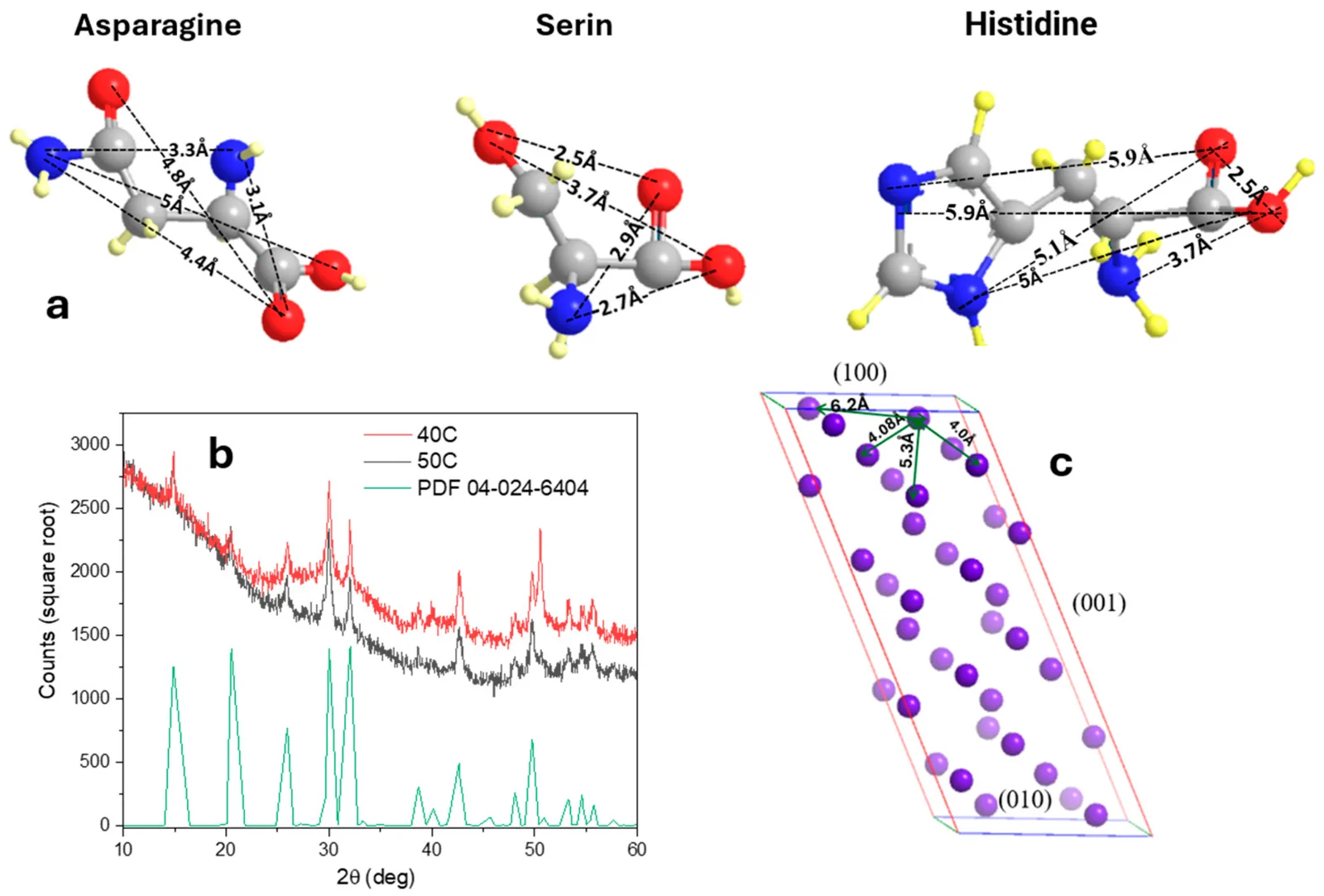
(**a**) Oxygen–oxygen and oxygen–nitrogen separations in Asn, Ser, and His, estimated by the ChemDraw software 2025 version (in solution we expect some distance flexibility). Red—oxygen, blue—nitrogen, gray—carbon, and yellow—hydrogen atoms. (**b**) XRD patterns of TbPO_4_·H_2_O NCs synthesized at 50 °C (black) and 40 °C (red). The bottom curve is the reference pattern for monoclinic Tb_3_(PO_4_)_3_(H_2_O)_2_ (PDF 04-024-6404), confirming that the NCs adopt the monoclinic (pseudo-hexagonal) structure with space group C2. (**c**) The unit cell arrangement of Tb^3+^ ions in the monoclinic structure with some near-neighbor distances between the ions. Only Tb^3+^ sites are shown for simplicity, since they are the important ones for coordination of oxygen and nitrogen atoms of the adsorbed molecules.

Interestingly, it was previously reported that the TbPO_4_·H_2_O NCs prepared with TA at 100 °C crystallize at the P3_1_21 or P3_2_21 hexagonal space group (see Figure S4). In the present work, the analysis of XRD patterns of NCs prepared at 40–50 °C better fit a monoclinic (pseudo-hexagonal) structure, corresponding to the (chiral) space group C2 (Figure 4b). Such lanthanide phosphate crystals are known to frequently alternate between these two phases [55], which probably have very close energies. TEM (see ref. [48]) and AFM (Supplementary Information Figure S5) images reveal that the NCs form very thin elongated prisms with typical dimensions of 0.4–1 µm × 10–40 nm × 2–20 nm. The atomic structure of the dominant (100) and (010) surfaces (>95% surface area) of the TbPO_4_·H_2_O NCs exposes Tb^3+^ ions with a nearest-neighbor separation of approximately 4.0–4.08 Å and a next-nearest-neighbor separation of approximately 5.3–5.6 Å (Figure 4c). Those distances are similar for both (100) and (010) facets, and very close to the ones found in the hexagonal phase [48]. The shortest intramolecular oxygen–oxygen and oxygen–nitrogen distances in Ser, Asn, and His, extracted from their crystal structures, fall in the range of 3.5–4.4 Å, suggesting that adjacent functional groups of these amino acids can engage simultaneously with neighboring Tb^3+^ surface sites separated by ~4.0 Å. Asn and His also have oxygen–oxygen and oxygen–nitrogen separation distances in the range of 5–6 Å, which suit the next-nearest-neighbor distances of surface Tb^3+^ sites. It should be noted that these intramolecular distances have a fairly high flexibility in the dissolved molecules and can thus accommodate a range of surface site separation distances.

This geometric complementarity enables simultaneous coordination of multiple functional groups with three surface Tb^3+^ ions, consistent with a three-point chiral recognition mechanism [48]. Such multi-point surface binding is expected to enhance both adsorption affinity and enable stereochemical discrimination, providing a structural rationale for the enantioselectivity observed for the amino acids used in this study. A parallel geometric relationship emerges for TA and Asp [48]. Additional support to the threepoint chiral recognition model was previously provided by the lack of adsorption enantioselectivity on the terbium phosphate surfaces in a molecular analog of TA with only two functional groups, methylsuccinic acid [48]. In the present work, we further corroborated this observation by studying the adsorption of a racemic mixture of the amino acid phenylalanine on Λ-TbPO_4_·H_2_O NCs, which also showed no enantioselectivity (Figure S6). The latter has a phenyl side chain, which is expected to have negligible interaction with the Tb^3+^ sites; hence, only the amine and carboxyl groups of this molecule are expected to coordinate with those sites.

The dominance of the (100) and (010) facet family—comprising > 95% of the total NC surface area—further underscores the importance of this geometric matching [48]. The remaining surface area, located primarily at crystal edges, consists of (001) facets, which contribute minimally to overall adsorption. Consequently, enantioselective adsorption is expected to occur predominantly on the well-defined chiral (100) and (010) surfaces, where the spatial arrangement of Tb^3+^ sites is consistent and predictable across the NC ensemble. This structural uniformity contrasts markedly with quartz particles [32], which present multiple crystallographically distinct faces with varied and sometimes opposing enantioselective characteristics. The structural homogeneity of TbPO_4_·H_2_O NCs thus renders them a well-defined, high-surface-area chiral platform uniquely suited for fundamental studies of enantioselective adsorption and molecular recognition on inorganic surfaces [56].

## 3. Materials and Methods

### 3.1. Materials

Terbium chloride hexahydrate (99.9%) was purchased from Sigma-Aldrich (Rehovot, Israel). Sodium dibasic phosphate (≥99.0%), D-tartaric acid (99%), L-tartaric acid (≥99.5%), L-His (≥99.0%), D-His (≥99.0%), L-Ser (≥99.0%), D-Ser (≥99.0%), L-Asn (≥99.0%), and D-Asn (≥99.0%) were purchased from Sigma-Aldrich (Rehovot, Israel). Hydrochloric acid (32%) was purchased from BioLab (Jerusalem, Israel). Ultrapure water with a resistivity of 18.2 MΩ·cm was obtained from a Millipore Direct-Q3 UV purification (Millipore-Sigma, Rehovot, Israel) system and used throughout all experiments. Racemic solutions of His, Ser, and Asn were prepared by mixing the corresponding L- and D-enantiomer solutions in equal amounts. When necessary, minor adjustments to the concentration of one enantiomer were performed to obtain a zero CD signal, confirming the preparation of optically balanced racemic mixtures.

### 3.2. Synthesis of Enantiopure TbPO_4_·H_2_O NCs

Enantiopure TbPO_4_·H_2_O NC seed NCs were synthesized following the procedure reported by Schwartz et al. [57]. Briefly, 285 μL of a 100 mM terbium chloride solution adjusted to acidic conditions (pH ≈ 2) with 300 μL of a 100 mM L-/D-TA and preheated to 50 °C was added to 10 mL of ultrapure water, followed by the rapid addition of 600 μL of a 100 mM Na_2_HPO_4_ solution, also preheated to 50 °C, under continuous stirring. The reaction mixture was stirred for 1 h at 50 °C until a milky suspension was obtained, indicating NC formation. The resulting enantiopure NC seeds were denoted as Λ-NCs when synthesized using L-TA and Δ-NCs when synthesized using D-TA. For seed-mediated synthesis, 100 μL of the prepared NC suspension (from a total volume of approximately 11 mL, corresponding to ~1% of the synthesis volume) was used as the seed solution. Ligand-free enantiopure TbPO_4_·H_2_O NCs were prepared by repeating the same synthetic procedure without the addition of TA, while introducing the corresponding Λ- or Δ-NC seeds to direct the crystal growth. The obtained NCs were purified by centrifugation to remove residual dissolved species. Considering that the introduced seed solution contained only ~1% of the original TA amount, the estimated TA concentration during the ligand-free synthesis was approximately 0.1 mM. After the final purification, which removed nearly all dissolved residual TA, less than 10% of this amount was estimated to remain associated with the NCs, resulting in a negligible residual TA concentration significantly below 10 μM in the final purified NC samples.

### 3.3. Adsorption Experiments

Enantioselective adsorption experiments of Asn, Ser, and His on chiral surface TbPO_4_·H_2_O NCs were performed using purified (nearly) ligand-free NCs obtained from the seed-mediated synthesis. After purification, the precipitated NCs collected by centrifugation at 3300× *g* for 10 min were redispersed in 2 mL aqueous solutions containing either individual amino acid enantiomers L- or D-Asn, L- or D-Ser, and L- or D-His or the corresponding racemic mixtures at different concentrations. The resulting suspensions were stirred for 2 h at room temperature to reach adsorption equilibrium. The pH values of the amino acid solutions were approximately 6.5 for Asn, 5.5 for Ser, and 6.7 for His. After adsorption, the NCs were separated from the solution by centrifugation at 3300× *g* for 10 min, and the supernatants containing the remaining non-adsorbed amino acids were collected. The concentrations of free enantiomers in the supernatants were subsequently determined by CD spectroscopy (and titration in case of racemic mixtures).

### 3.4. Determination of Adsorbed Amino Acid Concentrations

The adsorbed equivalent concentrations of Asn, Ser, and His on the surfaces of chiral TbPO_4_·H_2_O NCs were determined at different initial amino acid concentrations. Calibration curves correlating amino acid enantiomer concentrations with CD peak intensities were established using the characteristic CD peaks at 202–204 nm for Asn and Ser, and 212 nm for His (Supplementary Information, Figure S1). CD spectra were recorded using an Applied Photophysics Chirascan CD spectrometer. After the adsorption experiments, the NC suspensions were centrifuged, and the supernatants were collected to determine the concentration of unadsorbed amino acid remaining in solution. For individual enantiomer adsorption experiments, the adsorbed equivalent concentration was calculated as the difference between the initial enantiomer concentration before adsorption and the free enantiomer concentration measured in the supernatant after adsorption, according to Equation (1) (Supplementary Information, Figure S7, Tables S2–S4). The lowest detectable concentrations using CD peak height estimates were of the order of 0.01 mM, but reliable adsorbed equivalent concentration evaluations required a minimal 0.1 mM concentration.

### 3.5. Enantiomeric Excess Determination from Racemate Adsorption Experiments

CD spectroscopy directly provides the difference in concentration between the adsorbed L- and D-enantiomers (see Figures S8–S10 and Tables S5–S7), but it does not independently yield their sum. Consequently, the combined adsorbed concentration (${\left[L\right]}_{ads}+{\left[D\right]}_{ads}$) was determined by acid–base titration of the Asn- and His-containing supernatants recovered after centrifugal separation of the TbPO_4_·H_2_O NC—adsorbate complexes. Representative titration curves for Asn and His are provided in Supplementary Information (see Figure S3 and Table S1). Ser *ee* was not analysed due to its weaker enantioselectivity.

Titrations were conducted using NaOH solutions at concentrations of 0.4, 1.0, 2.0, and 3.0 mM. The appropriate NaOH concentration for each sample was selected to match the initial Asn and His concentrations, ensuring adequate sensitivity and well-defined equivalence points. The equivalence point in each titration was identified from the peak in the first derivative of the titration curve with respect to added NaOH volume.

The total concentration of unadsorbed Asn and His enantiomers in the supernatant was calculated by referencing the equivalence-point titrated volume (*V_free_*) obtained for each post-adsorption sample against that measured for the initial racemic solution before the adsorption experiment (*V_ref_*):

*C*_*free*_ = *C*_*initial*_ × (*V*_*free*_/*V*_*ref*_) (6)

Here, *C_free_* denotes the total molar concentration of both enantiomers remaining unadsorbed in the supernatant; *C_initial_* is the known concentration of the racemic Asn and His solutions prepared prior to the adsorption experiment.

The adsorbed Asn and His concentrations, representing the combined surface-bound amounts of both enantiomers, were subsequently obtained by mass balance:

*C*_*ads*_ = *[L]*_*ads*_ + *[D]*_*ads*_ = *C*_*initial*_ − *C*_*free*_ (7)

With the total adsorbed concentration established from titration, and the enantiomeric concentration difference *([L]_ads_ − [D]_ads_)* extracted from the CD spectra using the calibration curves, the adsorbed enantiomeric excess was calculated, as shown in Equation (4).

## 4. Conclusions

This study demonstrates that intrinsically chiral Λ- and Δ-TbPO_4_·H_2_O NCs enantioselectively adsorb Asn, Ser and His, extending previous findings on TA and ASP to a broader set of amino acids with diverse side-chain functionality. Single-enantiomer adsorption followed Langmuir behavior at low adsorbed equivalent concentration, with L-enantiomers preferentially binding Λ-NCs and D-enantiomers preferentially binding Δ-NCs. At higher adsorbed concentrations, the Langmuir model failed and the results were well described by the Frumkin model, which accounts for lateral attraction between the molecules adsorbed at neighboring sites. *ee* of adsorbed enantiomer mixtures from initial racemic mixtures was the highest at low racemate concentrations and declined with their increase. The close geometric match between Tb^3+^–Tb^3+^ spacings on the dominant (100) and (010) facets and the intramolecular functional-group distances within the amino acid molecules, together with the lack of enantioselectivity for amino acids with only two coordinating groups, such as phenylalanine, supports a three-point chiral recognition mechanism as the structural basis for this enantioselectivity. Taken together with earlier results for TA and ASP, these findings establish a universal relationship between molecular functional-group geometry and enantioselective adsorption strength on chiral NC surfaces. We believe that the high enantioselectivity observed for the NCs at low adsorbate concentrations could be exploited for asymmetric heterogeneous catalysis.

## Figures and Tables

**Table 1:** Adsorption equilibrium constants *K* and maximum adsorption capacities *Q* obtained from Langmuir model fitting for the L- and D-enantiomers of Ser, Asn, His, Asp and TA adsorbed onto Λ- and Δ-TbPO_4_·H_2_O NCs.

Amino Acid	Parameter	L on Λ-NC	L on Δ-NC	D on Δ-NC	D on Λ-NC
Ser	*K* (mM^−1^)	0.053 ± 0.01	0.027 ± 0.01	0.058 ± 0.01	0.024 ± 0.006
	*Q* (mM)	4.8 ± 1.2	4.9 ± 1.7	4.2 ± 0.9	4.7 ± 1.2
Asn	*K* (mM^−1^)	0.12 ± 0.06	0.071 ± 0.03	0.12 ± 0.01	0.068 ± 0.02
	*Q* (mM)	2.8 ± 0.3	1.9 ± 0.7	2.9 ± 0.3	1.9 ± 0.6
His	*K* (mM^−1^)	0.15 ± 0.01	0.079 ± 0.01	0.16 ± 0.01	0.075 ± 0.009
	*Q* (mM)	1.49 ± 0.07	1.42 ± 0.15	1.44 ± 0.09	1.51 ± 0.16
Asp *	*K* (mM^−1^)	0.20 ± 0.04	0.10 ± 0.01	0.21 ± 0.04	0.12 ± 0.02
	*Q* (mM)	2.0 ± 0.1	2.1 ± 0.5	1.9 ± 0.2	2.0 ± 0.3
TA *	*K* (mM^−1^)	0.10 ± 0.03	0.05 ± 0.03	0.10 ± 0.02	0.04 ± 0.02
	*Q* (mM)	2.5 ± 0.4	2.8 ± 0.7	2.6 ± 0.4	2.9 ± 0.6

* Values taken from Ref. [48].

**Table 2:** Adsorption equilibrium constants *K*, inter-molecular interaction *U* and maximum adsorption capacities *Q* obtained from Frumkin model fitting for the L- and D-enantiomers of His and Asn adsorbed onto Λ- and Δ-TbPO_4_·H_2_O NCs.

Amino Acid	Parameter	L on Λ-NCs	D on Λ-NCs	D on Δ-NCs	L on Δ-NCs
His	*K* (mM^−1^)	0.121 ± 0.003	0.054 ± 0.004	0.118 ± 0.008	0.052 ± 0.002
	*Q* (mM)	1.553 ± 0.002	1.521 ± 0.005	1.562 ± 0.006	1.527 ± 0.003
	*U*	−0.74 ± 0.05	−1.84 ± 0.11	−0.71 ± 0.11	−1.84 ± 0.07
Asn	*K* (mM^−1^)	0.097 ± 0.015	0.047 ± 0.006		
	*Q* (mM)	1.828 ± 0.004	1.932 ± 0.011		
	*U*	−2.17 ± 0.19	−2.02 ± 0.17		

## Data Availability

The original contributions presented in this study are included in the article/Supplementary Material. Further inquiries can be directed to the corresponding author(s).

## Supplementary Materials

The following supporting information can be downloaded at https://www.mdpi.com/article/10.3390/molecules31183187/s1: Figure S1: Calibration curves for CD peak intensities of Asn, Ser, and His as a function of known enantiomer concentrations. Figure S2: The adsorption of single Asn enantiomers (in terms of equivalent adsorbed concentration) to Λ- and Δ-NCs as a function of the free Asn, Ser, and His enantiomer concentration. Figure S3: Potentiometric titration curves used to calculate free L+D His/Asn concentrations at different starting racemate concentrations. Table S1: Summarizes the adsorption analysis for racemic His and Asn mixtures on the NCs, including the total initial racemate concentration, the free (unadsorbed) L+D concentration determined by potentiometric titration. Figure S4: Powder XRD pattern of TbPO4·H2O NCs synthesized with TA at 100 °C. Figure S5: AFM images of TbPO4·H2O NCs. Figure S6: CD spectra of pre- and post-adsorption of phenylalanine. Figure S7: displays the CD spectra of adsorbed L- and D-Asn at different starting concentrations (indicated in each graph) after adsorption on Δ- or Λ-NCs. Table S2: CD-derived free and adsorbed equivalent concentrations of Asn enantiomers on Λ- and Δ-TbPO_4_·H_2_O NCs. Table S3: shows the equivalent amounts of Ser enantiomers on Λ- and Δ-TbPO_4_·H_2_O NCs, as determined by CD. Table S4: shows the CD peak values and estimated free and equivalent adsorption concentrations of His single enantiomers for varying starting concentrations. Figure S8: Raw CD spectra from adsorption experiments of racemic Ser at varied initial concentrations to either Λ- or Δ-NCs. Table S5: CD and concentration differential data from rac-Ser adsorption on NCs. Figure S9: Raw CD spectra from adsorption investigations with rac-Asn mixtures at varying concentrations and Λ- or Δ-NCs. Table S6: CD and concentration difference data from the rac-Asn adsorption on NCs. Figure S10: Raw CD spectra from adsorption experiments with racemic His mixtures at varying concentrations and Δ- or Λ-NCs. Table S7: presents CD and concentration differential data from rac-His adsorption on NCs.
