# Supplementary material for: Universal Behavior in Enantioselective Adsorption of Amino Acids onto Chiral Terbium Phosphate Nanocrystals
Source: Molecules. 2026 Sep 10;31(18):3187. doi: 10.3390/molecules31183187 (PMC13609759; doi:10.3390/molecules31183187)
Supplement: Supplementary file 1 [file molecules-31-03187-s001.zip › molecules-4514811-supplementary.pdf]

Supplementary Information for:

Universal behaviour in enantioselective adsorption of amino acids onto chiral terbium phosphate nanocrystals

Abdullah Idrees and Gil Markovich*

Circular Dichroism Analysis for Enantioselective Adsorption of asparagine, serine, and histidine on Chiral Surfaces of $TbPO_4 \cdot H_2O$ NCs.

To analyse the enantioselective adsorption of amino acids on the chiral surfaces of $TbPO_4 \cdot H_2O$ NCs, a calibration curve of the circular dichroism (CD) signal as a function of concentration was created for each amino acid enantiomer pair.

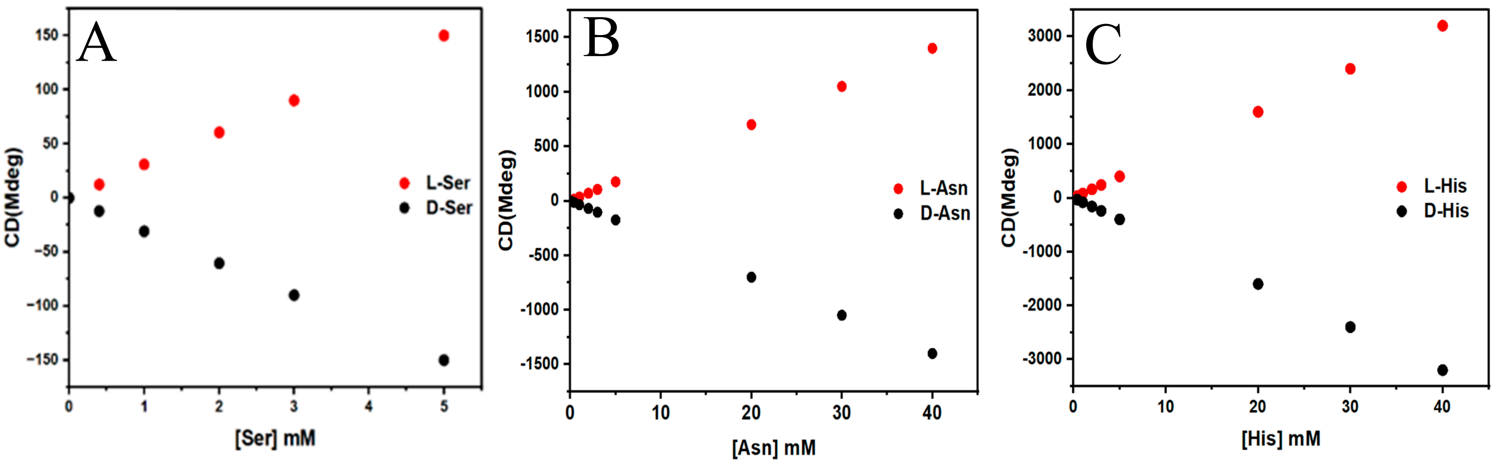

Figure S1. Calibration curves for CD peak intensities of Ser, Asn, and His as a function of known enantiomer concentrations. (A) CD peak heights recorded at 202-204 nm in aqueous solutions of L-Ser and D-Ser at concentrations ranging from 0 to 5 mM. (B) CD peak heights measured at 202-204 nm in aqueous L-Asn and D-Asn solutions at concentrations ranging from 0 to 40 mM. (C) CD peak heights were determined at 212 nm for aqueous L-His and D-His solutions at concentrations ranging from 0 to 40 mM.

Enantioselective adsorption Langmuir isotherms of Asn, Ser, and His on chiral $TbPO_4{\cdot}H_2O$ NCs

Figure S2. Individual-enantiomer adsorption isotherms of Asn, Ser, and His at relatively low surface coverage on Λ- and Δ-$TbPO_4{\cdot}H_2O$ NCs. The adsorbed equivalent concentration (C~ads~) is plotted as a function of the equilibrium free enantiomer concentration (C~free~) for all four enantiomer–nanocrystal chirality combinations L- and D-enantiomer on Λ- and Δ-NCs. Symbols show experimental data; solid lines represent fits to the Langmuir adsorption model Equation 2. In each panel, the matched chiral pairs L-enantiomer on Λ-NCs and D-enantiomer on Δ-NCs define the upper curve, while the mismatched pairs (L-enantiomer on Δ-NCs and D-enantiomer on Λ-NCs) define the lower curve. (a) Asn; (b) Ser; (c) His.

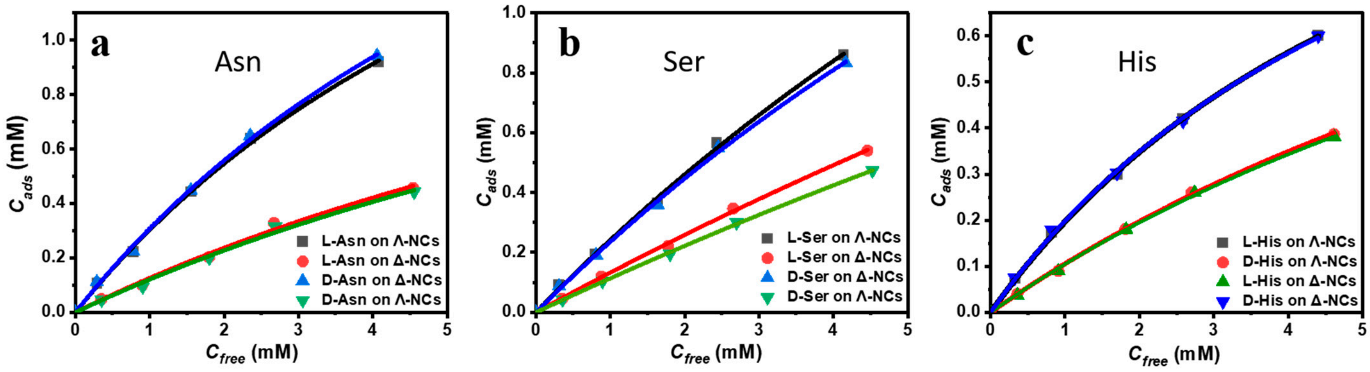

Figure S2. (a) The adsorption of single Asn enantiomers (in terms of equivalent adsorbed concentration) to Λ- and Δ-NCs as a function of the free Asn enantiomer concentration. The symbols show experimental data, whereas the lines represent the fitted Langmuir curves. (b) Same for Ser. (c) the same for His.

Potentiometric titration curves are used to calculate the equivalent adsorbed concentration of L+D molecules in racemate adsorption investigations.

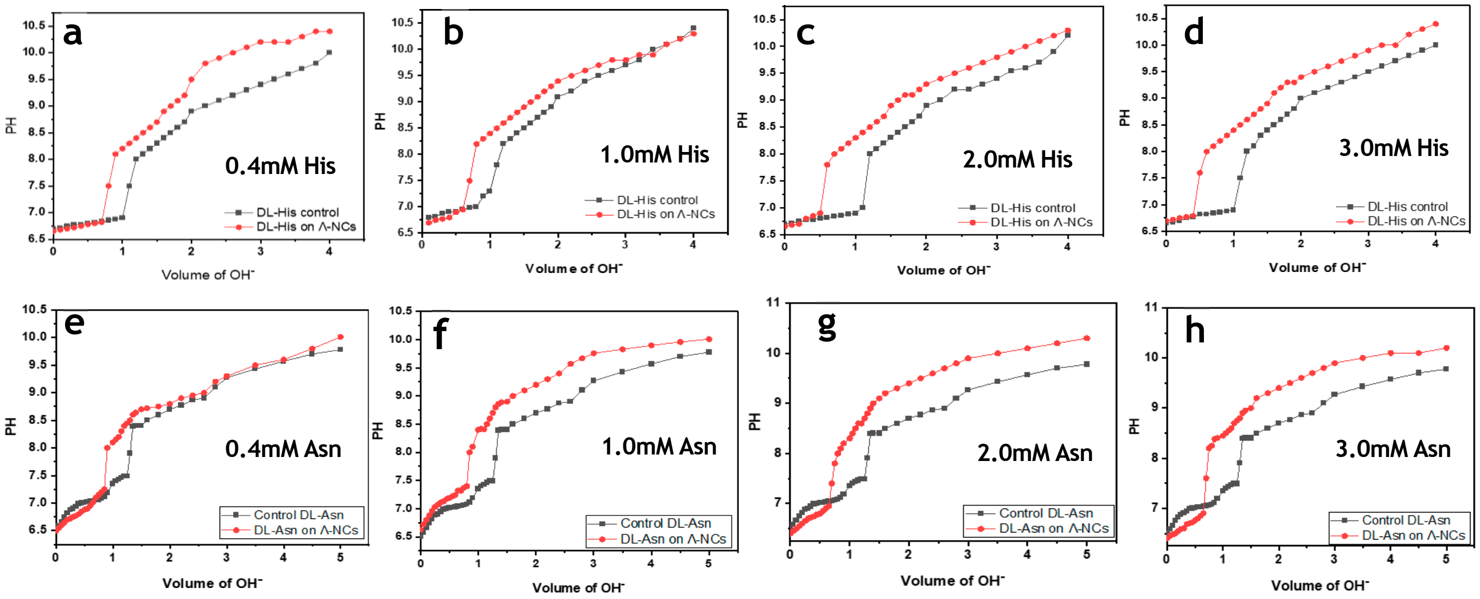

Figure S3. (a-d) Potentiometric titration curves were used to calculate free L+D-His concentrations at different starting racemate concentrations. The control samples included 2 mL of rac-His solutions (black) and free (unadsorbed) L+D-His molecules following adsorption from the racemate solution onto Λ-NCs and subsequent separation (red). Both sets of solutions were titrated with NaOH at the same beginning concentrations (0.4,1,2,3 mM). (e-h) Potentiometric titration curves were used to calculate free L+D-Asn concentrations at different starting racemate concentrations. Control samples contained 2 mL of rac-Asn solutions (black) and free (unadsorbed) L+D-Asn molecules after adsorption from racemate solution onto Λ-NCs and separation (red). Both sets of solutions were titrated with NaOH solutions having concentrations equal to the initial concentrations of the amino acid racemates pre-adsorption (0.4, 1.0, 2.0 ,3.0 mM).

The peaks of the first-derivative curves were used to represent equivalence points. Equivalence points for post-adsorption titrations depicting the free, unadsorbed acid concentration were calculated relative to the control sample. For more information, see the Experiment Section.

Adsorbed *ee* values estimated for adsorption experiments from racemic mixtures

Table S1 presents concentration data from adsorption experiments conducted with racemic His and Asn mixtures on NCs for initial racemate concentrations of 0.4, 1.0, 2.0, 3.0 mM. Adsorbed equivalent concentrations for both amino acids gradually increased with increasing initial concentration.

Table S1. summarizes the adsorption analysis for racemic His and Asn mixtures on the NCs, including the total initial racemate concentration, the free (unadsorbed) L+D concentration determined by potentiometric titration, the adsorbed equivalent concentration of $C_{ads} = [L]_{ads} + [D]_{ads} = C_{initial} - C_{free}$, and the estimated enantiomeric excess of the adsorbed fraction: $ee(\%) = 100 \times |\frac{[L]_{ads}-[D]_{ads}}{[L]_{ads}+[D]_{ads}}|$

**[L-His] + [D-His] (mM)**				
Total (initial) concentration (mM)	0.4	1.0	2.0	3.0
Free acid concentration (mM)	0.29	0.64	1.05	1.36
Total adsorbed equivalent concentration (mM)	0.109	0.36	0.95	1.64
ee (%) $[L]_{ads}$-$[D]_{ads}$ obtained from CD used data from table S7	**32**	**13.8**	**7.7**	**6**
**[L-Asn] + [D-Asn] (mM)**				
Total (initial) concentration (mM)	0.4	1.0	2.0	3.0
Free acid concentration (mM)	0.28	0.65	1.11	1.61
Total adsorbed equivalent concentration (mM)	0.12	0.35	0.89	1.39
ee (%) $[L]_{ads}$-$[D]_{ads}$ obtained from CD used data from table S6	**22.5**	**11**	**5.3**	**3.9**

X-ray Powder Diffractometry (XRD)

The XRD pattern of the $TbPO_4 \cdot H_2O$ NCs synthesized with TA at 100°C shows broadened peaks that match well with the hexagonal $GdPO_4 \cdot H_2O$ reference, used since a reference pattern for hexagonal $TbPO_4 \cdot H_2O$ is not available in the database. This confirms the hexagonal structure (space group $P3_121$ or $P3_221$) for the NCs produced at 100°C.

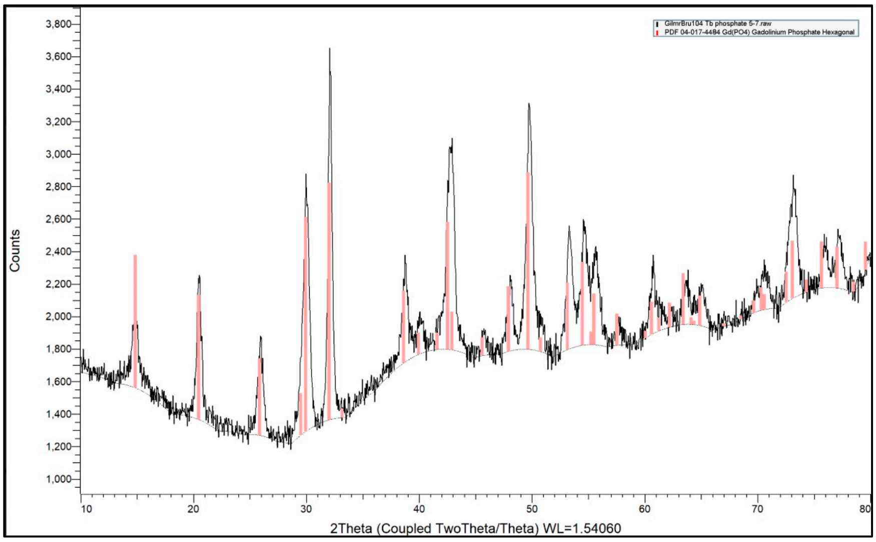

Figure S4. Powder XRD pattern of $TbPO_4 \cdot H_2O$ NCs synthesized with TA at 100°C (black), matched against the reference pattern of hexagonal $GdPO_4$ (PDF 04-017-4484, red), confirming the hexagonal structure space group $P3_121$ or $P3_221$.

Atomic Force Microscopy (AFM)

AFM imaging of the $TbPO_4 \cdot H_2O$ NCs (Figure S3) reveals very thin, elongated prismatic structures, with height profiles indicating typical dimensions of 0.4 -1 µm × 10 - 40 nm × 2 - 20 nm.

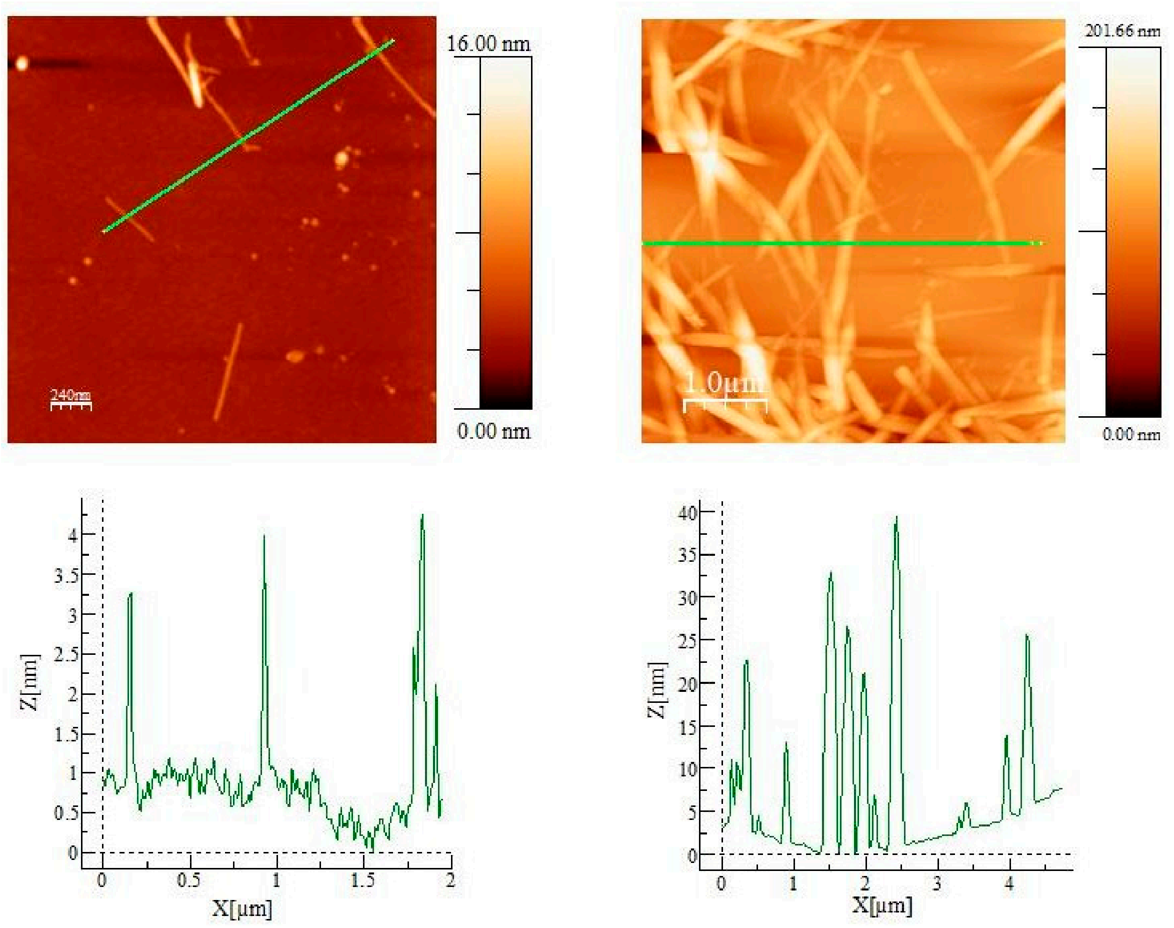

Figure S5. AFM images of $TbPO_4 \cdot H_2O$ NCs, with two height profiles of the NCs, revealing very thin elongated prisms with typical dimensions of 0.4 - 1 µm × 10 - 40 nm × 2 - 20 nm.

Adsorption of rac-phenylalanine on Λ-NCs

The amino acid phenylalanine has only two functional groups (amine, carboxyl) which can coordinate with the surface Tb^{3+} sites, while the phenyl side group is not expected to significantly interact with those sites. Hence, a test of enantioselectivity in their adsorption on the terbium phosphate would help prove or disprove the three-point chiral recognition model that we propose here. As can be seen in Figure S6 below, adsorption of a racemic mixture of phenylalanine enantiomers on Λ-NCs results in zero enantioselectivity, which is very different from the other amino acids studied in this work.

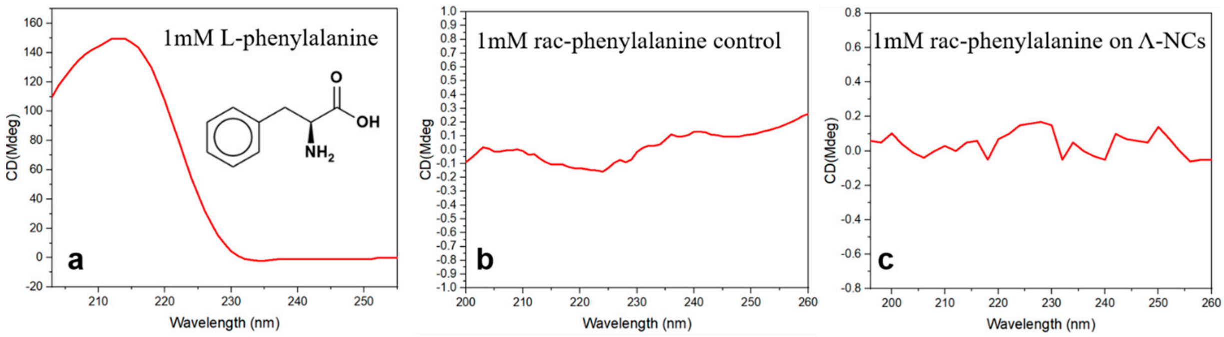

Figure S6. (a) CD spectrum of 1 mM L-phenylalanine. (b) CD spectrum of 1 mM of a racemic mixture of phenylalanine enantiomers before the adsorption experiment. (c) CD spectrum of the supernatant with free phenylalanine enantiomers after separating out the Ncs + adsorbed molecules after an adsorption experiment with the racemic phenylalanine mixture of 1 mM. The absence of a detectable CD peak at 215 nm indicates lack of enantioselective adsorption. This is very different from the results with the other three amino acids studied: His, Ser Asn (see Figures S8-10 below).

Raw CD data following adsorption of single enantiomers of Asn onto the NCs

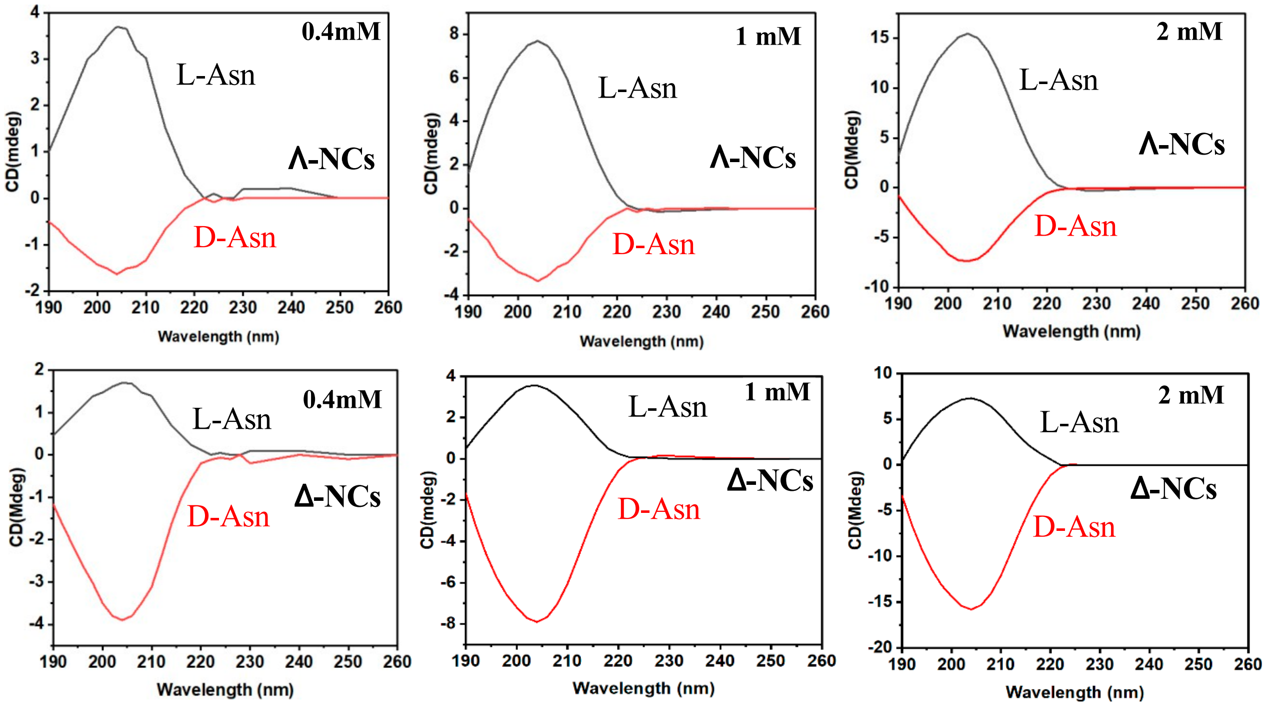

Figure S7. displays the CD spectra of adsorbed L- and D-Asn at different starting concentrations (indicated in each graph) after adsorption on Δ- or Λ-NCs. After each adsorption experiment, the NCs and adsorbed molecules were separated by centrifugation, and the supernatant solution was measured. The amount adsorbed was calculated by subtracting the unadsorbed (free) concentration from the initial concentration. Concentration values were determined using the CD signal at 202-204 nm and the calibration curves in Fig. S1. These findings were used to construct the chart shown in Figure 1a.

Similar findings were obtained for Ser and His, as evidenced by the calibration curves and adsorption data presented in Tables S3 and S4.

Table S2. CD-derived free and adsorbed equivalent concentrations of Asn enantiomers on Λ- and Δ-$TbPO_4·H_2O$ NCs.

L/D Asn initial enantiomer concentration (mM)	Adsorbed enantiomer CD peak value at 202-204 nm (Mdeg) after adsorption to Λ-NCs	Adsorbed enantiomer CD peak value at 202-204 nm (Mdeg) after adsorption to Δ-NCs	Free enantiomer concentration (mM)		Adsorbed equivalent concentration (mM)	
			Λ-NCs	Δ-NCs	Λ-NCs	Δ-NCs
L-Asn (0.4)	3.7	1.7	0.29	0.35	0.10	0.04
D-Asn (0.4)	-1.6	-3.9	0.35	0.28	0.04	0.11
L-Asn (1.0)	7.8	3.5	0.77	0.90	0.22	0.10
D-Asn (1.0)	-3.3	-7.9	0.90	0.77	0.09	0.22
L-Asn (2.0)	15.5	7.1	1.55	1.79	0.44	0.20
D-Asn (2.0)	-7	-15.7	1.8	1.55	0.20	0.44
L-Asn (3.0)	22.3	11.5	2.36	2.67	0.63	0.32
D-Asn (3.0)	-11	-22.7	2.68	2.35	0.31	0.64
L-Asn (5.0)	32.2	16	4,14	4.54	0,86	0.45
D-Asn (5.0)	-15.5	-33	4.55	4.05	0.44	0.94
L-Asn (20)	59.5	-	18.2	-	1.7	-
D-Asn (20)	-56	-	18.4	-	1.6	-
L-Asn (30)	61.5	-	28.25	-	1.75	-
D-Asn (30)	-60	-	28.28	-	1.71	-
L-Asn (400)	62	-	38.23	-	1.77	-
D-Asn (400)	-62.3	-	38.22	-	1.78	-

Table S3. shows the equivalent amounts of Ser enantiomers on Λ- and Δ-$TbPO_4·H_2O$ NCs, as determined by CD.

L/D Ser Enantiomer Initial Conc. (mM)	Adsorbed enantiomer CD peak 202-204 nm (Mdeg) after ads. to Λ-NCs	Adsorbed enantiomer CD peak 202-204 nm (Mdeg) after ads. to Δ-NCs	Free conc. (mM)		Adsorbed equiv. conc. (mM)	
			Λ-NCs	Δ-NCs	Λ-NCs	Δ-NCs
L-Ser (0.4)	2.8	1.4	0.30	0.35	0.09	0.04
D-Ser (0.4)	-1.3	-2.7	0.35	0.31	0.04	0.087
L-Ser (1.0)	6	3.7	0.80	0.88	0.19	0.11
D-Ser (1.0)	-3.2	-5.9	0.89	0.80	0.10	0.19
L-Ser (2.0)	11	6.7	1.63	1.77	0.36	0.22
D-Ser (2.0)	-5.9	-10.8	1.8	1.64	0.19	0.35
L-Ser (3.0)	17	10.4	2.43	2.65	0.56	0.34
D-Ser (3.0)	-9	-16.5	2.7	2.45	0.30	0.55
L-Ser (5.0)	25.8	16.2	4.14	4.46	0.86	0.54
D-Ser (5.0)	-14.2	-25	4.52	4.16	0.47	0.83

Table S4. shows the CD peak values and estimated free and equivalent adsorption concentrations of His single enantiomers for varying starting concentrations.

L/D His Enantiomer Initial Conc. (mM)	Adsorbed enantiomer CD peak 212 nm (Mdeg) after ads. to Λ-NCs	Adsorbed enantiomer CD peak 212 nm (Mdeg) after ads. to Δ-NCs	Free conc. (mM)		Adsorbed equiv. conc. (mM)	
			Λ-NCs	Δ-NCs	Λ-NCs	Δ-NCs
L-His (0.4)	5.9	3	0.32	0.36	0.073	0.037
D-His (0.4)	-3.2	-6	0.36	0.32	0.040	0.075
L-His (1.0)	13.9	7.3	0.82	0.91	0.17	0.09
D-His (1.0)	-7.2	-14.3	0.91	0.82	0.09	0.178
L-His (2.0)	24	14.4	1.7	1.82	0.30	0.18
D-His (2.0)	-14.5	-24.2	1.81	1.69	0.18	0.30
L-His (3.0)	33.6	20.8	2.58	2.74	0.42	0.26
D-His (3.0)	-21	-33.2	2.7	2.58	0.26	0.41
L-His (5.0)	48.2	30.6	4.4	4.62	0.60	0.38
D-His (5.0)	-30.6	-48	4.61	4.4	0.38	0.6
L-His (20)	100	100	18.75	18.75	1.25	1.25
D-His (20)	-101	-99.4	18.74	18.76	1.26	1.24
L-His (30)	108	108	28.65	28.65	1.35	1.35
D-His (30)	-108	-107.8	28.65	28.6	1.35	1.35
L-His (40)	112	112	38.6	38.6	1.40	1.40
D-His (40)	-112	-112	38.6	38.6	1.40	1.40

Raw CD data for adsorption experiments of racemic Ser:

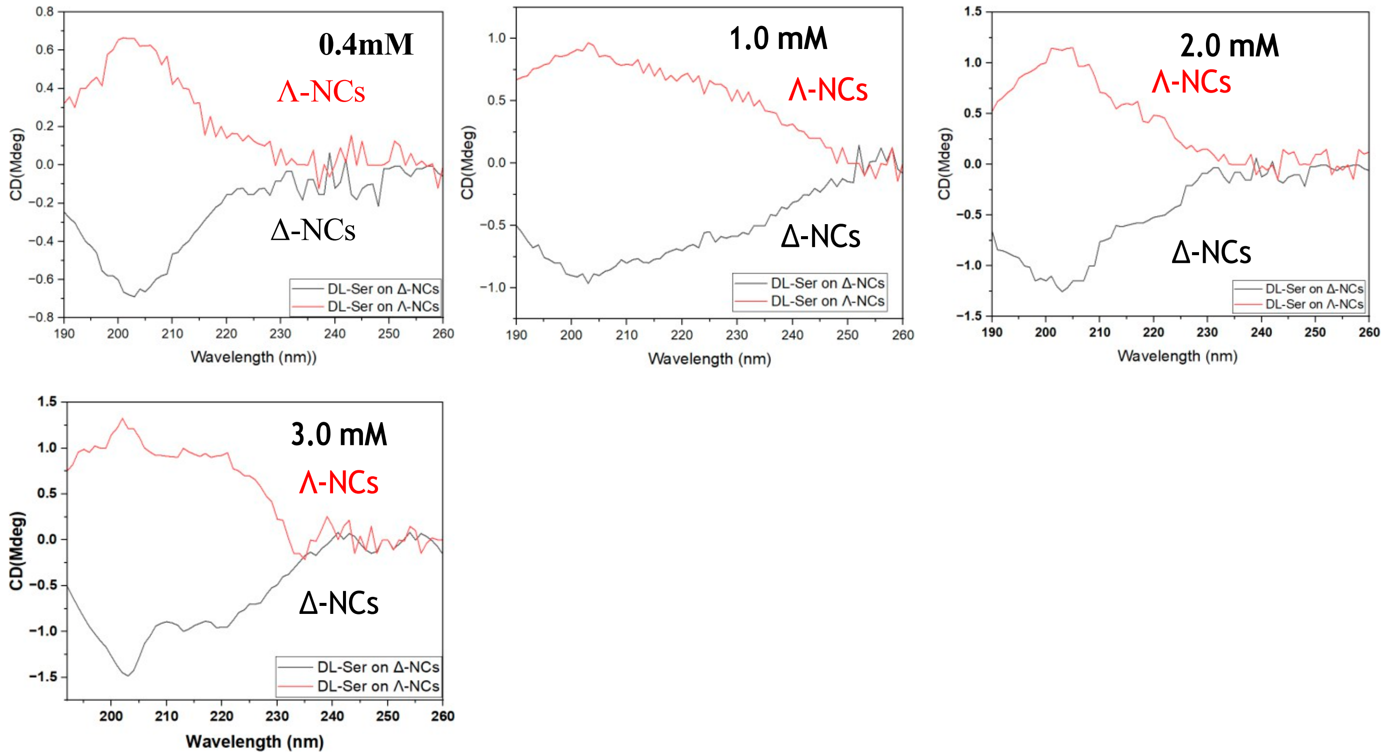

Figure S8. Raw CD spectra from adsorption experiments of racemic Ser at varied initial concentrations to either Λ- or Δ-NCs. The measurements are for free, unadsorbed molecules following centrifugation to remove adsorbed molecules and NCs. The CD peaks represent the differences between free D and L enantiomer concentrations. Thus, the difference in adsorbed equivalent concentration is L - D (the CD peak × -1). The peak CD values occur at 202 - 204 nm.

Table S5. CD and concentration differential data from rac-Ser adsorption on NCs.

Initial rac-Ser concentration (mM)	CD peak of free molecules after adsorption to Δ-NCs (mdeg)	CD peak of free molecules after adsorption to Λ-NCs (mdeg)	Enantiomer concentration difference averaged over Λ- and Δ-NCs (mM)
0.4	-0.65	0.64	0.021
1.0	-0.90	0.91	0.029
2.0	-1.17	1.14	0.038
3.0	-1.45	1.31	0.045

Raw CD data for adsorption experiments of racemic Asn:

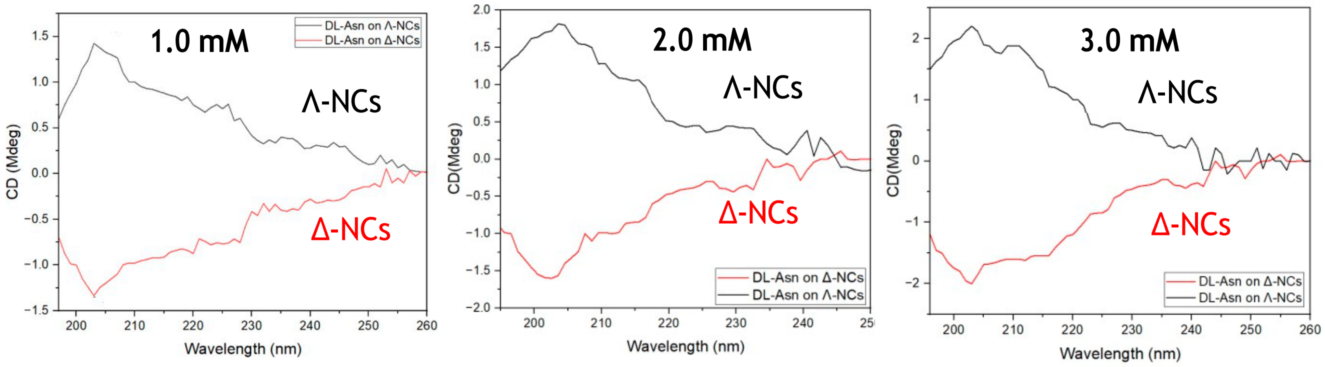

Figure S9. Raw CD spectra from adsorption investigations with rac-Asn mixtures at varying concentrations and Λ- or Δ-NCs. After centrifugation, spectra were obtained from the residual free (unadsorbed) fraction. The recorded CD signal corresponds to the D-L concentration difference in the free fraction; hence the equivalent concentration difference of the adsorbed fraction is L-D (the CD peak ×-1). The peak CD values at 202-204 nm were obtained.

Table S6. CD and concentration difference data from the rac-Asn adsorption on NCs.

Initial Rac-Asn Concentration (mM)	CD peak of free molecules after adsorption to Δ-NCs (mdeg)	CD peak of free molecules after adsorption to Λ-NCs (mdeg)	Enantiomer concentration difference averaged over Λ- and Δ-NCs (mM)
0.4	-0.92	1.06	0.027
1.0	-1.33	1.42	0.039
2.0	-1.61	1.81	0.048
3.0	-1.84	2.04	0.055

Raw CD data for adsorption experiments of racemic His:

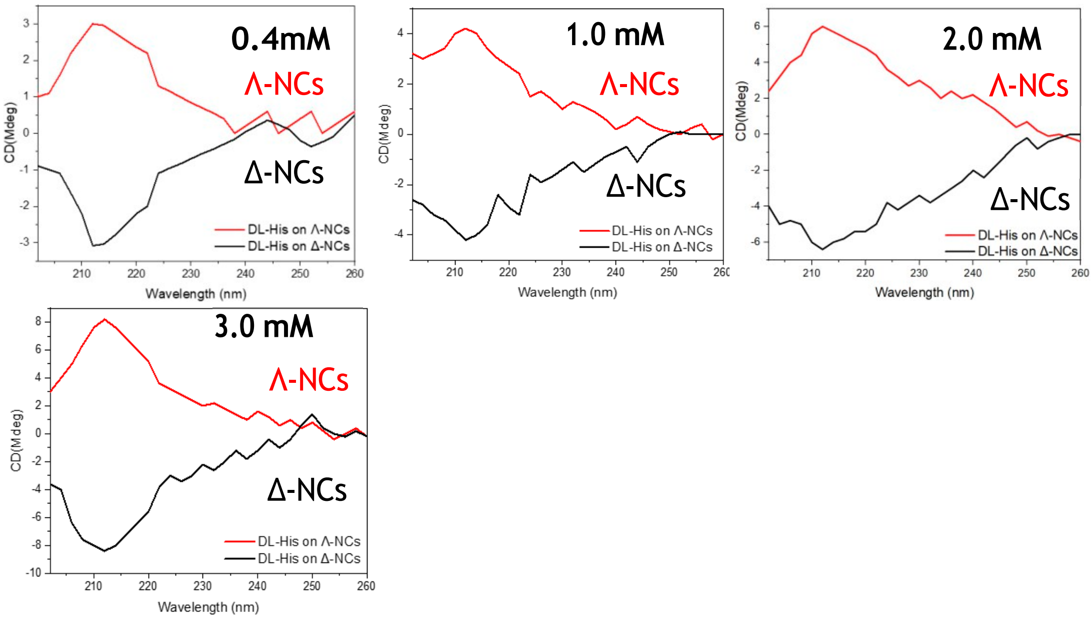

Figure S10. Raw CD spectra from adsorption experiments with racemic His mixtures at varying concentrations and Δ- or Λ-NCs. After centrifugation, spectra were obtained from the residual free (unadsorbed) fraction. Because the recorded CD signal represents the D - L concentration difference in this free fraction, the analogous concentration difference of the adsorbed fraction is L – D (the CD peak × -1). Peak CD values at 212 nm were obtained.

Table S7. presents CD and concentration differential data from rac-His adsorption on NCs.

Initial Rac-His Concentration (mM)	CD peak of free molecules after adsorption to Δ-NCs (mdeg)	CD peak of free molecules after adsorption to Λ-NCs (mdeg)	Enantiomer concentration difference averaged over Λ- and Δ-NCs (mM)
0.4	-2.77	2.85	0.035
1.0	-4	4.06	0.050
2.0	-6.13	5.76	0.074
3.0	-8.13	7.8	0.099
